# Proposal of a Novel Risk Score for Determination of Coronary Artery Disease Risk in Newly Diagnosed Diabetes Patients

**DOI:** 10.18502/ijph.v49i8.3907

**Published:** 2020-08

**Authors:** Cosmin Mihai VESA, Carmen RADU, Loredana POPA, Claudia JURCA, Lucia DAINA, Cornelia BALA, Gabriela ROMAN, Mădălina MOISI, Amorin POPA, Mihaela POPOVICIU, Anca FERICIAN, Dana ZAHA

**Affiliations:** 1. Faculty of Medicine and Pharmacy, University of Oradea, Oradea, Romania; 2. Clinical County Emergency, Hospital of Oradea, Oradea, Romania; 3. Faculty of Medicine, University of Medicine and Pharmacy “Iuliu Hațieganu”, Cluj-Napoca, Romania

## Dear Editor-in-Chief

Coronary artery disease (CAD) is the leading cause of death worldwide (1). Age is a major contributor to the alteration of vascular function, that even in the absence of conditions like hypertension or diabetes, age-associated modifications occur (2). The increasing percent of aging population is the explanation why CAD remains the leading cause of mortality even in countries with high standard of living where adequate public health measures have been taken for risk reduction.

Diabetes mellitus is a condition that has reached endemic proportions, in 2017 there were 424 million cases worldwide, over 90% of patients being type 2 diabetes mellitus cases (3). The connection between diabetes mellitus and CAD is the severity of atherosclerosis generated by insulin resistance and all is pathophysiological consequences: hyperglycaemia, hypertension, dyslipidaemia, increased arterial stiffness, sympathetic system activation. Diabetes mellitus patients have 2 to 4 fold increase of coronary artery disease morbidity (4), the prevalence of hypertension in diabetic individuals is estimated to be around 80% and the prevalence of dyslipidaemia is estimated to be around 70% (5).

Therefore, we investigated the possibility of finding a mathematical formula that can combine these two major risk factors: age and insulin resistance for the accurate CAD risk prediction at 10 years.

We investigated our hypothesis on 121 newly diagnosed patients with diabetes mellitus. All patients gave their consent for participation in the study. Patients were selected from the population of newly diagnosed diabetes mellitus cases from Bihor County, Romania from 2017. For each of them the determination of coronary artery disease morbidity risk at 10 years was performed using the UK Prospective Diabetes Study (UKPDS) risk engine with calculation of coronary heart disease (CHD) component.

To assess the efficacy of our score proposed score, named Cardiovascular Assessment of Morbidity (CVAMO) Score, it was mandatory to take UKPDS risk engine score as a validation instrument, to calculate the statistical significance of the association between our score, CVAMO, and UKPDS risk engine score. To determine the CAD component of UKPDS risk engine for every patient we determined the following variables: age, sex, presence of atrial fibrillation, smoking status, systolic blood pressure, glycated haemoglobin (HbA1c), total cholesterol and HDL- cholesterol. It can be easily observed that UKPDS risk engine has many variables. Actually, it is composed of 10 variables, the 8 ones enumerated above and diabetes duration, which was 0 because the patients were newly diagnosed, and ethnicity.

**Fig. 1:**
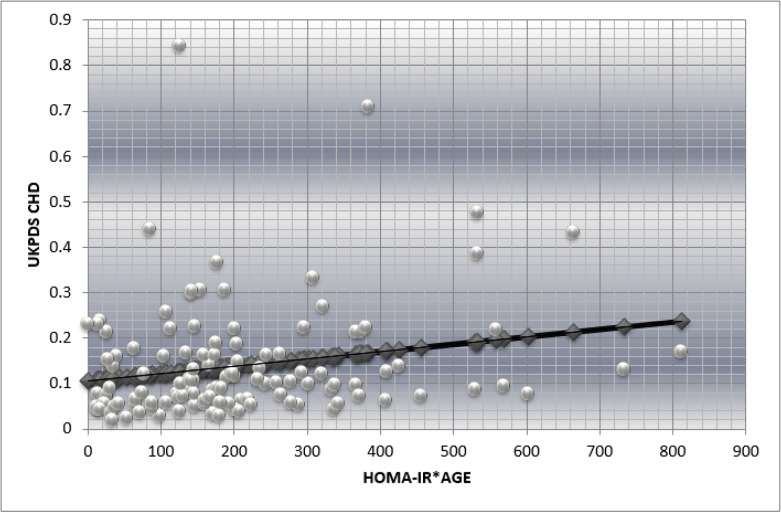
Linear correlation between insulin resistance multiplied by age of the 121 patients and UKPDS risk engine scores of patients for the prediction of CAD morbidity at 10 years

The score we propose is a simpler one composed of only two variables the age of the patient and insulin resistance as assessed by Homeostatic Model Assessment for Insulin Resistance (HOMA-IR) index. The formula used for HOMA-IR index was the standard international formula: HOMA-IR = (FPI×FPG)/22.5, where FPI=fasting plasma insulin and FPG=fasting plasma glucose. The construction of score was a 2-step process: firstly, we calculated the correlation between insulin resistance and UKPDS CHD score. Then we multiplied the age of the patients with insulin-resistance and correlated the product with the results obtained using UKPDS risk engine score. We obtained a statically significant correlation (*P*<0.05) between the product HOMA-IR*age and UKPDS risk engine score (Fig. 1, Table 1). The linear regression equation between HOMA-IR*age and UKPDS CHD component constitutes the formula of our score.

**Table 1:** Statistical data concerning association between CVAMO and UKPDS

***Variable***	***Coefficient***	***Standard Error***	***Lower Confidence Limit***	***Upper Confidence Limit***	***t Stat***	***P***
Intercept	0.10	0.01	0.06	0.14	5.7	6.1667E-8
HOMA-IR*AGE	0.0002	6.9900E-5	2.4312E-5	0.0003	2.3	0.02

The equation of our score is the following: CVAMO (%) = (0.10 + 0.0002 * HOMA-IR*AGE) * 100, Were CVAMO is the risk of a newly diagnosed patient for suffering a major coronary artery disease event at 10 years from diabetes diagnosis. Our score is easy to apply in clinical practice, HOMA-IR determination is generally available and with lower costs than the determination of glycated haemoglobin, cholesterol and non-HDL-cholesterol and EKG performance for determination of presence of atrial fibrillation. Our findings also demonstrate that insulin resistance and age have multiplicative effects on the risk of cardiovascular morbidity, supporting their causative role in events such as myocardial infarction or unstable angina. The category of patients with ages <65 yr old and non-insulin resistant (HOMA-IR <2.5) had a mean CVAMO score of 8.51%, while the category of patients with ages >65 years old and insulin resistant (HOMA-IR≥2.5) had a mean CVAMO score of 17.65%, statistically significant higher than the first category (*P*<0.01).

CVAMO score is a novel proposed score for coronary artery disease risk assessment at 10 yr of newly diagnosed diabetes patients. For its determination, the physician needs to know only the severity of insulin resistance of the patient with the help of HOMA-IR index and of course, the patient’s age. Determination of cardiovascular disease risk is very important since it represents the leading cause of death worldwide, and insulin resistance can be easily modified by simple measurements such as diet adjustment with lowering of body mass index.
